# Synergistic Effect of Ambient PM_2.5_ Exposure and Waist-Hip Ratio on Non-Alcoholic Fatty Liver Disease Risk in a Taiwanese Population

**DOI:** 10.7150/ijms.126734

**Published:** 2026-03-30

**Authors:** Shih-Tsung Chang, Ying-Hsiang Chou, Chih-Da Wu, Oswald Ndi Nfor, Wen Yu Lu, Chien-Ning Huang, Yung-Po Liaw

**Affiliations:** 1 Institute of Medicine, Chung Shan Medical University, Taichung City, 40201, Taiwan.; 2 Department of Radiation Oncology, Chung Shan Medical University Hospital, Taichung City, 40201, Taiwan.; 3 School of Medicine, Chung Shan Medical University, Taichung City,40201, Taiwan.; 4 School of Medical Imaging and Radiological Sciences, Chung Shan Medical University, Taichung City, 40201, Taiwan.; 5 National Institute of Environmental Health Sciences, National Health Research Institutes, Miaoli, Taiwan.; 6 Department of Geomatics, National Cheng Kung University, Tainan, Taiwan.; 7 Department of Public Health, Institute of Public Health, Chung Shan Medical University, Taichung City 40201, Taiwan.; 8 Department of Internal Medicine, Chung Shan Medical University Hospital, Taichung City, 40201, Taiwan.; 9 Department of Medical Imaging, Chung Shan Medical University Hospital, Taichung City 40201, Taiwan.; 10 Department of Public Health, College of Public Health, China Medical University, 406040, No. 100, Sec. 1, Jingmao Rd., Beitun Dist., Taichung, Taiwan.; 11 Department of Medical Research, China Medical University Hospital, China Medical University, Taichung City, Taiwan.

**Keywords:** PM_2.5_ exposure, waist-hip ratio, non-alcoholic fatty liver disease, land-use regression model

## Abstract

Epidemiological studies have established a connection between environmental factors and the prevalence of non-alcoholic fatty liver disease (NAFLD). Fine particulate matter with an aerodynamic diameter ≤ 2.5 μm (PM_2.5_), a major component of ambient air pollution, has been implicated in systemic inflammation and metabolic dysfunction. The waist-hip ratio (WHR), an important measure of body composition, is a key risk factor for metabolic syndrome and NAFLD. We investigated the association between ambient PM_2.5_ exposure, WHR, and NAFLD. We used data from the Taiwan Biobank collected between 2016 and 2020, involving 15,049 individuals aged 30 to 70 years. Ambient PM_2.5_ exposure was estimated using a land-use regression model and categorized into quartiles. NAFLD was identified using liver function tests and imaging, and WHR was classified as normal or abnormal based on sex-specific cutoffs. Participants with NAFLD (n = 7179) had higher body mass index, a greater prevalence of abnormal WHR, and less favorable lipid profiles compared with those without NAFLD (n = 7870). Higher PM_2.5_ exposure was associated with increased odds of NAFLD, with participants in the highest quartile (PM_2.5_ > 39.85 μg/m³) showing an odds ratio (OR) of 1.251 (95% CI: 1.114-1.404). An abnormal WHR was also associated with higher odds of NAFLD (OR = 1.641, 95% CI: 1.507-1.787). A statistically significant interaction between PM_2.5_ exposure and WHR was observed, with individuals with abnormal WHR exhibiting consistently higher odds of NAFLD across all PM_2.5_ quartiles. In the highest PM_2.5_ quartile, participants with abnormal WHR had an OR of 2.020 (95% CI: 1.708-2.389), compared with an OR of 1.442 (95% CI: 1.215-1.712) among those with normal WHR. These findings suggest that central obesity may amplify the association between PM_2.5_ exposure and NAFLD, highlighting the importance of integrated public health strategies targeting both air pollution and metabolic health.

## Introduction

Air pollution and obesity are significant public health issues that have a profound impact on metabolic disorders worldwide. PM_2.5_ is particularly harmful because its small size allows it to penetrate deeply into the lungs and enter the systemic circulation, thereby contributing to respiratory and cardiovascular diseases. Increasing evidence also suggests that PM_2.5_ exposure may play an essential role in metabolic dysregulation, including the development of NAFLD 1, 2.

Epidemiological studies have increasingly linked environmental factors to the rising global prevalence of NAFLD. Among these factors, PM_2.5_ has gained attention due to its adverse effects on liver health. VoPham et al. (2022) 3 and Guo et al. (2022) 4 reported that PM_2.5_ exposure is associated with an increased risk of metabolic dysfunction-associated fatty liver disease (MAFLD), potentially mediated through systemic inflammation and oxidative stress that promote hepatic steatosis and fibrosis. Additional epidemiological evidence further supports the association between PM_2.5_ exposure and NAFLD risk 5.

Given that PM_2.5_ exposure contributes to metabolic dysfunction, individual susceptibility factors—particularly those related to body fat distribution—may modify its impact on liver disease risk. WHR, an important indicator of central adiposity, is a well-established risk factor for metabolic syndrome and NAFLD 6-8. Emerging evidence suggests that central obesity may exacerbate the deleterious effects of PM_2.5_ on liver health. Excess visceral fat promotes oxidative stress, chronic inflammation, and adipocytokine imbalance, mechanisms that may amplify PM_2.5_-induced hepatic injury and accelerate NAFLD development 9, 10.

In Taiwan, where the prevalence of NAFLD has been steadily increasing 11, the Taiwan Biobank (TWB) provides a valuable population-based resource for examining the interplay between environmental exposures, anthropometric measures, and metabolic diseases. Previous studies, including those by Yang et al. (2021) 2 have leveraged TWB data to identify associations among environmental factors, genetic predispositions, lifestyle characteristics, and liver disease risk.

Building on this growing body of evidence, the present study aimed to investigate the synergistic effect of ambient PM_2.5_ exposure and WHR on NAFLD prevalence in a Taiwanese population. By integrating air quality data with biometric and health information from the TWB, we sought to provide a comprehensive assessment of how environmental and metabolic factors jointly influence NAFLD risk. Understanding these interactions is particularly important in densely populated regions such as Taiwan, where industrial pollution remains a critical public health concern. Ultimately, our findings may inform targeted prevention strategies and public health policies addressing both environmental and metabolic determinants of NAFLD. Accordingly, we analyzed TWB data collected between 2016 and 2020 from 15,049 participants aged 30-70 years to examine the relationship between ambient PM_2.5_ exposure, WHR, and NAFLD prevalence.

## Materials and Methods

### Study design and participants

This study employed a cross-sectional design using baseline data from the TWB collected between 2016 and 2020. Participants were community-dwelling adults aged 30-70 years who completed baseline health examinations, including waist and hip circumference measurements, and had available PM_2.5_ exposure estimates. Individuals assessed in different enrollment years represent independent participants, and no longitudinal follow-up or repeated measurements were conducted. Participants with incomplete data were excluded, resulting in a final analytical sample of 15,049 subjects.

The study protocol was approved by the institutional review committee of Chung Shan Medical University (CS1-22209, CS1-20009, and CS1-23101). All TWB participants provided written informed consent at enrollment.

### Exposure assessment and outcome measures

The TWB does not directly collect air pollution data. Air pollution metrics, including PM_2.5_ concentrations, were obtained from the Taiwan Environmental Protection Administration (EPA), which operates approximately 71 automated monitoring stations across Taiwan and records daily average concentrations of various pollutants. For this study, PM_2.5_ concentrations from 2000 to 2016 were used to characterize long-term ambient exposure based on participants' residential locations, reflecting cumulative environmental exposure before health assessment.

Annual average PM_2.5_ concentrations, expressed in micrograms per cubic meter (μg/m³), were derived from daily measurements. To account for spatial and temporal variability across Taiwan, PM_2.5_ exposure levels were estimated for 349 geographic areas using a machine learning-coupled land-use regression (LUR) model, as described previously 12. Participants were assigned PM_2.5_ exposure estimates based on their residential locations.

We acknowledge that the temporal gap between the end of the exposure period (2016) and health assessments for some participants (up to 2020) may raise concerns. Accordingly, the PM_2.5_ exposure metric was defined to reflect historical long-term exposure rather than contemporaneous PM_2.5_ levels, consistent with prior epidemiological studies examining the chronic health effects of air pollution. This approach assumes that cumulative exposure to ambient PM_2.5_ contributes to long-term metabolic disturbances, including the development of NAFLD.

PM_2.5_ exposure levels were categorized into quartiles as follows: PM_2.5_ ≤ Q1 (PM_2.5_ ≤ 28.90 μg/m^3^), Q1 < PM_2.5_ ≤ Q2 (28.90 < PM_2.5_ ≤ 35.99 μg/m^3^), Q2 < PM_2.5_ ≤ Q3 (35.99 < PM_2.5_ ≤ 39.85 μg/m^3^), and PM_2.5_ > Q3 (PM_2.5_ > 39.85 μg/m^3^).

NAFLD status was determined using liver enzyme measurements and imaging data (e.g., ultrasound) available in the TWB. Potential NAFLD cases were identified based on abnormal liver enzyme values or imaging evidence of hepatic steatosis. Self-reported disease information in the biobank reflects physician-diagnosed conditions.

Waist circumference was measured at the narrowest point between the ribs and iliac crest, and hip circumference at the widest point over the buttocks. WHR was calculated by dividing waist circumference by hip circumference. WHR was classified as normal or abnormal according to sex-specific cutoffs: ≤ 0.90 for men and ≤ 0.85 for women were considered normal, while values above these thresholds were considered abnormal.

### Covariates

Covariate data were collected using standardized questionnaires and clinical measurements and included age, sex, smoking status, exercise habits, body mass index (BMI), vegetarian diet, coffee consumption, alanine aminotransferase/aspartate aminotransferase (ALT/AST) ratio, total cholesterol, triglycerides, high-density lipoprotein cholesterol (HDL-C), and low-density lipoprotein cholesterol (LDL-C). These variables were included as potential confounders based on prior literature and biological plausibility.

### Statistical analysis

All statistical analyses were conducted using SAS software version 9.4. Participant characteristics were summarized using chi-square tests for categorical variables and Student's t-tests for continuous variables. Logistic regression models were used to estimate crude and multivariable-adjusted ORs and 95% CIs for the associations between PM_2.5_ exposure, WHR, and NAFLD.

Crude models estimated unadjusted associations by including each exposure variable of interest (e.g., PM_2.5_ quartiles or WHR) separately without additional covariates. Multivariable-adjusted logistic regression models were subsequently constructed to control for potential confounding factors selected a priori based on previous literature and biological plausibility. These covariates (detailed above) are predefined risk factors for NAFLD and may also be associated with exposure to air pollution or body fat distribution.

BMI and WHR were included simultaneously to distinguish overall adiposity from central fat distribution. PM_2.5_ exposure was categorized into quartiles, with the lowest quartile serving as the reference group. Effect modification by WHR was evaluated by including a multiplicative interaction term between PM_2.5_ exposure and WHR. Stratified analyses by PM_2.5_ quartiles were also performed to examine the association between WHR and NAFLD across different exposure levels.

## Results

### Demographic and baseline characteristics

The study included 15,049 participants, divided into two groups based on the presence of NAFLD: 7870 participants without NAFLD and 7179 with NAFLD (Table 1). Significant differences were observed between the two groups across various demographic and physiological measures. Participants with NAFLD exhibited a higher body mass index (BMI) (26.089 ± 0.044 vs. 22.844 ± 0.034, p < 0.0001), a higher WHR (70.71% with an abnormal ratio vs. 40.00% in the non-NAFLD group, p < 0.0001), and altered lipid profiles, including higher triglycerides (152.600 ± 1.397 vs. 93.982 ± 0.630, p < 0.0001) and lower high-density lipoprotein (49.634 ± 0.136 vs. 59.030 ± 0.154, p < 0.0001). The prevalence of NAFLD was also higher among older adults (73.38% aged ≥ 50 vs. 66.25% in the non-NAFLD group, p < 0.0001) and men (36.51% vs. 25.24%, p < 0.0001).

### Association of PM_2.5_ exposure and waist-hip ratio with NAFLD

In univariate analyses (Table 2), higher PM_2.5_ exposure was associated with an increased likelihood of NAFLD. Participants in the highest PM_2.5_ quartile (PM_2.5_ > Q3) had a significantly higher crude odds of NAFLD compared with those in the lowest quartile (OR = 1.251, 95% CI: 1.114-1.404, p < 0.0001), with a significant trend across quartiles (p-trend < 0.0001). Similarly, an abnormal WHR was associated with higher odds of NAFLD (OR = 1.641, 95% CI: 1.507-1.787, p < 0.0001) compared with a normal ratio. Crude analyses also indicated that men had lower odds of NAFLD than women (OR = 0.848, 95% CI: 0.765-0.940, p = 0.0017), and participants aged ≥ 50 years had higher odds compared with those < 50 years (OR = 1.438, 95% CI: 1.306-1.584, p < 0.0001). Lifestyle factors, including smoking and exercise, were not significantly associated with NAFLD in these univariate analyses.

A statistically significant interaction between PM_2.5_ exposure and WHR on the odds of NAFLD was observed (p for interaction = 0.0388). In stratified multivariable-adjusted analyses by PM_2.5_ quartiles (Table 3), an abnormal WHR was consistently associated with higher odds of NAFLD across all levels of PM_2.5_ exposure. The adjusted odds ratios for abnormal versus normal WHR were 1.737 (95% CI: 1.448-2.084) for PM_2.5_ ≤ Q1, 1.636 (95% CI: 1.383-1.935) for Q1 < PM_2.5_ ≤ Q2, 1.730 (95% CI: 1.465-2.044) for Q2 < PM_2.5_ ≤ Q3, and 1.508 (95% CI: 1.275-1.783) for PM_2.5_ > Q3.

Joint exposure analyses further supported this interaction (Table 4). After adjustment for potential confounders, participants with abnormal WHR and PM_2.5_ > Q3 had the highest odds of NAFLD (OR = 2.020, 95% CI: 1.708-2.389) compared with the reference group (normal WHR and PM_2.5_ ≤ Q1). In contrast, among participants with normal WHR, higher PM_2.5_ exposure was associated with a more modest increase in NAFLD odds (OR = 1.442, 95% CI: 1.215-1.712).

## Discussion

Our study found a significant interaction between ambient PM_2.5_ levels and WHR in the risk of developing NAFLD among adults in Taiwan. Individuals with an abnormal WHR coupled with higher quartiles of PM_2.5_ exposure displayed an increased propensity for NAFLD. Notably, the highest quartile of PM_2.5_ exposure was associated with a marked increase in NAFLD risk, particularly among those with an abnormal WHR. These findings suggest that increased central adiposity, as indicated by WHR, may exacerbate the hepatotoxic effects of air pollution, aligning with broader evidence on the health impacts of particulate matter 13.

The higher risk for NAFLD among individuals with abnormal WHR and higher levels of PM_2.5_ may be attributed to several potential mechanisms. Firstly, abnormal WHR, indicative of central obesity, is often associated with adipose tissue dysfunction, which can disrupt lipid metabolism and result in increased release of free fatty acids (FFAs) into the bloodstream. These FFAs can be taken up by the liver, promoting hepatic fat accumulation. This aligns with previous studies highlighting the role of adipose tissue dysfunction in NAFLD development 14, 15. Secondly, exposure to higher levels of PM_2.5_ has been linked to systemic inflammation and oxidative stress 16. Inflammatory mediators and oxidative stress can induce liver injury and inflammation, contributing to the development and progression of NAFLD 17, 18. Furthermore, abnormal WHR is often associated with insulin resistance, a key factor in the pathogenesis of NAFLD. Exposure to PM_2.5_ has been shown to exacerbate insulin resistance and impair glucose homeostasis, further contributing to liver fat accumulation and NAFLD development 19, 20.

Regarding the specific association between PM_2.5_, WHR, and NAFLD, there is limited prior evidence focusing on WHR as a modifier of PM_2.5_-related NAFLD risk. While several studies have examined the impact of general obesity on the health effects of air pollution, few have specifically investigated how central obesity, as measured by WHR, influences these effects. Our study adds a novel dimension to the existing literature by highlighting the synergistic contribution of central adiposity and air pollution to NAFLD risk.

Beyond its hepatic consequences, NAFLD is increasingly recognized as a multisystem disease, with cardiovascular disease representing its most common and clinically significant comorbidity. Central obesity and metabolic dysfunction are key contributors to both NAFLD and cardiovascular risk. Environmental exposures such as ambient PM_2.5_ may exacerbate these risks by promoting systemic inflammation and atherosclerosis, thereby linking liver and vascular pathology. Indeed, chronic PM_2.5_ exposure has been strongly associated with cardiovascular disease incidence and mortality, highlighting its systemic impact 21. Evidence suggests that NAFLD severity correlates with early markers of atherosclerosis, such as carotid intima-media thickness (IMT). For example, a study by Tarantino et al. 22 demonstrated that carotid IMT is predicted by combined eotaxin levels and hepatic steatosis severity in obese patients with NAFLD, suggesting a direct link between liver fat accumulation and vascular changes. Our findings of synergistic effects between PM_2.5_ and central obesity on NAFLD risk may therefore have broader implications, potentially contributing to the increased cardiovascular risk observed in individuals with NAFLD, particularly in populations exposed to high levels of air pollution.

Taken together, our findings suggest that the combination of abnormal WHR and higher levels of PM_2.5_ may synergistically contribute to an increased risk for NAFLD. Adipose tissue dysfunction, inflammation, oxidative stress, and insulin resistance may serve as underlying mechanisms linking these factors. These findings have significant clinical and public health implications. Clinically, individuals with abnormal WHR and higher levels of PM_2.5_ may benefit from specific interventions. Lifestyle modifications, such as regular exercise programs and weight management strategies, could be recommended to mitigate NAFLD risk in these individuals. Additionally, counseling on measures to reduce exposure to air pollution, such as avoiding high-pollution areas and using air purifiers, may also be beneficial.

A multidisciplinary approach is crucial to address the complex relationship between abnormal WHR, PM_2.5_, and NAFLD. Collaboration between healthcare providers, public health agencies, and environmental organizations is essential to develop comprehensive strategies that address both individual and environmental risk factors, ultimately contributing to the prevention of NAFLD and improvement of population health.

One of the strengths of our study is the use of a large, well-characterized cohort from the TWB, which allowed for a detailed assessment of both PM_2.5_ exposure and health outcomes. The application of LUR models for estimating PM_2.5_ exposure also adds precision to our exposure assessment (Huang et al., 2019). However, our study has limitations. The cross-sectional design limits our ability to establish causality between PM_2.5_ exposure, WHR, and NAFLD. Furthermore, reliance on self-reported data for some variables may introduce reporting bias. Lastly, despite adjustment for several potential confounders, residual confounding by unmeasured factors, such as dietary intake and genetic predispositions, cannot be ruled out. Nevertheless, our findings provide valuable insights into the clinical and public health implications of the association between abnormal WHR, PM_2.5_, and NAFLD, and lay the groundwork for future investigations in this area.

## Conclusion

In summary, this study underscores the significant correlation between higher levels of PM_2.5_ exposure and the increased risk of NAFLD, with the greatest risk observed in individuals in the highest quartile of exposure. The consistent elevation in NAFLD risk among individuals with an abnormal WHR across all levels of PM_2.5_ exposure highlights the critical interplay between environmental and metabolic factors. These findings support integrated public health strategies that address both air pollution and metabolic health to mitigate the burden of NAFLD and its associated cardiovascular complications. Future research should continue to explore these interactions to develop more targeted and effective prevention and management strategies.

## Figures and Tables

**Table 1:** Demographic, lifestyle, and clinical characteristics of participants with and without non-alcoholic fatty liver disease

Variables	No NAFLD	NAFLD	p-value
	(n = 7870)	(n = 7179)	
Mean PM_2.5_ (μg/m^3^), %			0.3603
PM_2.5_ ≤ Q1	1714 (21.78)	1591 (22.16)	
Q1 < PM_2.5_ ≤ Q2	2085 (26.49)	1871 (26.06)	
Q2 < PM_2.5_ ≤ Q3	2184 (27.75)	1925 (26.81)	
PM_2.5_ > Q3	1887 (23.98)	1792 (24.96)	
Waist-hip ratio, n (%)			< 0.0001
Normal	4722 (60.00)	2103 (29.29)	
Abnormal	3148 (40.00)	5076 (70.71)	
Gender, n (%)			< 0.0001
Women	5884 (74.76)	4558 (63.49)	
Men	1986 (25.24)	2621 (36.51)	
Age, n (%)			< 0.0001
Age < 50	2656 (33.75)	1911 (26.62)	
Age ≥ 50	5214 (66.25)	5268 (73.38)	
Smoking status, n (%)			< 0.0001
Non-smoker	6998 (88.92)	5962 (83.05)	
Current smoker	872 (11.08)	1217 (16.95)	
Exercise, n (%)			< 0.0001
No	4044 (51.39)	3936 (54.83)	
Yes	3826 (48.61)	3243 (45.17)	
BMI, (kg/m^2^)	22.844 ± 0.034	26.089 ± 0.044	< 0.0001
Vegetarian, n (%)			0.3179
No	7137 (90.69)	6544 (91.15)	
Yes	733 (9.31)	635 (8.85)	
Coffee consumption, n (%)			0.0727
No	4603 (58.49)	4095 (57.04)	
Yes	3267 (41.51)	3084 (42.96)	
ALT/AST ratio	0.763 ± 0.003	1.022 ± 0.004	< 0.0001
Total Cholesterol	196.200 ± 0.397	199.900 ± 0.442	< 0.0001
Triglyceride	93.982 ± 0.630	152.600 ± 1.397	< 0.0001
High-density lipoprotein	59.030 ± 0.154	49.634 ± 0.136	< 0.0001
Low-density lipoprotein	118.600 ± 0.347	125.300 ± 0.394	< 0.0001

Abbreviations: NAFLD, nonalcoholic fatty liver disease; PM, particulate matter; n, sample size; %, percent; BMI, body mass index; kg, kilogram; m^2^, square meters; ALT, alanine aminotransferase; AST, aspartate aminotransferase.

**Table 2:** Univariate (crude) logistic regression analyses examining the associations between PM_2.5_ exposure, WHR, and other individual factors with odds of NAFLD.

Variables	OR	95% CI	p-value
Mean PM_2.5_			
PM_2.5_ ≤ Q1 (ref)	-	-	-
Q1 < PM_2.5_ ≤ Q2	0.964	0.860-1.080	0.5223
Q2 < PM_2.5_ ≤ Q3	1.034	0.923-1.158	0.5625
PM_2.5_ > Q3	1.251	1.114-1.404	0.0001
p-trend	P-value < 0.0001		
Waist-hip ratio			
Normal (ref)	-	-	-
Abnormal	1.641	1.507-1.787	< 0.0001
Sex			
Women (ref)	-	-	-
Men	0.848	0.765-0.940	0.0017
Age			
Age < 50 (ref)	-	-	-
Age ≥ 50	1.438	1.306-1.584	< 0.0001
Smoking status			
Non-smoker (ref)	-	-	-
Current smoker	0.917	0.806-1.044	0.1913
Exercise			
No (ref)	-	-	-
Yes	1.001	0.922-1.087	0.9804
BMI	1.189	1.172-1.205	< 0.0001
Vegetarian diet			
No (ref)	-	-	-
Yes	0.941	0.820-1.080	0.3860
Coffee consumption			
No (ref)	-	-	-
Yes	1.069	0.897-1.158	0.0997
ALT/AST ratio	6.866	5.898-7.994	< 0.0001
Total Cholesterol	0.980	0.975-0.986	< 0.0001
Triglyceride	1.010	1.009-1.011	< 0.0001
High-density lipoprotein	1.001	0.994-1.008	0.7423
Low-density lipoprotein	1.023	1.017-1.028	< 0.0001
interaction: PM_2.5_*waist-hip ratio	p-value = 0.0388		

OR, odds ratio; CI, confidence interval; ref, reference; BMI, body mass index; ALT, alanine aminotransferase; AST, aspartate aminotransferase; WHR, waist-hip ratio.

**Table 3:** Associations between WHR and odds of NAFLD stratified by PM_2.5_ quartiles, estimated using multivariable-adjusted logistic regression models.

Variables	PM_2.5_ ≤ Q1			Q1 < PM_2.5_ ≤ Q2			Q2 < PM_2.5_ ≤ Q3			PM_2.5_ > Q3		
	OR	95% CI	p-value	OR	95% CI	p-value	OR	95% CI	p-value	OR	95% CI	p-value
Waist-hip ratio												
Normal (ref)	-	-	-	-	-	-	-	-	-	-	-	-
Abnormal	1.737	1.448-2.084	<0.0001	1.636	1.383-1.935	<0.0001	1.730	1.465-2.044	<0.0001	1.508	1.275-1.783	< 0.0001
Sex												
Women (ref)	-	-	-	-	-	-	-	-	-	-	-	-
Men	0.928	0.744-1.157	0.5065	0.803	0.656-0.984	0.0341	0.906	0.744-1.104	0.3275	0.778	0.633-0.957	0.0176
Age												
Age < 50 (ref)	-	-	-	-	-	-	-	-	-	-	-	-
Age ≥ 50	1.485	1.190-1.854	0.0005	1.453	1.206-1.750	< 0.0001	1.441	1.199-1.732	< 0.0001	1.376	1.136-1.667	0.0011

OR, odds ratio; CI, confidence interval; ref, reference; BMI, body mass index; ALT, alanine aminotransferase; AST, aspartate aminotransferase; WHR, waist-hip ratio. Adjusted for smoking status, exercise, BMI, diet, coffee intake, ALT, Total cholesterol, HDL-C, LDL-C, and Triglycerides.

**Table 4:** Joint associations of WHR and PM_2.5_ exposure quartiles with odds of NAFLD, estimated using multivariable-adjusted logistic regression models

Variables	OR	95% CI	P-value
Waist-hip ratio and mean PM_2.5_			
Normal waist-hip ratio, PM_2.5_ ≤ Q1 (ref)	-	-	-
Normal waist-hip ratio, Q1 < PM_2.5_ ≤ Q2	1.010	0.848-1.204	0.9098
Normal waist-hip ratio, Q2 < PM_2.5_ ≤ Q3	1.064	0.894-1.265	0.4843
Normal waist-hip ratio, PM_2.5_ > Q3	1.442	1.215-1.712	<0.0001
Abnormal waist-hip ratio, PM_2.5_ ≤ Q1	1.819	1.531-2.160	<0.0001
Abnormal waist-hip ratio, Q1 < PM_2.5_ ≤ Q2	1.685	1.432-1.984	<0.0001
Abnormal waist-hip ratio, Q2 < PM_2.5_ ≤ Q3	1.833	1.558-2.157	<0.0001
Abnormal waist-hip ratio, PM_2.5_ > Q3	2.020	1.708-2.389	<0.0001

Abbreviation: OR, odds ratio; CI, confidence interval; ref, reference; BMI, body mass index; ALT, alanine aminotransferase; AST, aspartate aminotransferase; WHR, waist-hip ratio. Adjusted for age, sex, smoking, exercise, BMI, diet, coffee intake, ALT, Total cholesterol, HDL-C, LDL-C, and Triglycerides.

## Data Availability

Data are available from the authors upon reasonable request and with permission of the Taiwan Biobank.

## Abbreviations

OR: odds ratio
CI: confidence interval
ref: reference
BMI: body mass index
ALT: alanine aminotransferase
AST: aspartate aminotransferase
WHR: waist-hip ratio
NAFLD: non-alcoholic fatty liver disease
PM: particulate matter
WHR: waist-hip ratio
